# Detection of Anti-*Neospora caninum* IgG in Blood Serum and Colostrum Samples in Naturally Infected Sheep and in Their Newborn Offspring

**DOI:** 10.3390/pathogens11111263

**Published:** 2022-10-29

**Authors:** Roberto Alves Bezerra, Brendo Andrade Lima, Felipe Boniedj Ventura Alvares, Gabriel Augusto Marques Rossi, Fabio Ribeiro Braga, Renata Pimentel Bandeira de Melo, Rinaldo Aparecido Mota, Vinícius Longo Ribeiro Vilela, Thais Ferreira Feitosa

**Affiliations:** 1 Laboratory of Immunology and Infectious Diseases, Department of Veterinary Medicine, Instituto Federal da Paraíba (IFPB), Sousa 58807-630, PA, Brazil; 2 Post-Graduating Program of Science and Animal Health, Universidade Federal de Campina Grande (UFCG), Patos 58708-110, PA, Brazil; 3 Laboratory of Experimental Parasitology, Department of Veterinary Medicine, Universidade Vila Velha (UVV), Vila Velha 29102-920, ES, Brazil; 4 Department of Veterinary Medicine, Universidade Federal Rural de Pernambuco (UFRPE), Recife 52171-900, PE, Brazil

**Keywords:** neosporosis, offspring, serology, ovine

## Abstract

The aim was to detect correlations of IgG antibodies against *N. caninum* in serum and colostrum samples from ewes, through the IFAT, and to evaluate the presence of this immunoglobulin in the serum of newborn lambs after colostrum ingestion. Blood samples from 162 ewes that did not show any disease in the general physical examination and from their newborn lambs, not more than five days postpartum, along with 162 colostrum samples and 182 blood samples from the neonates, were analyzed. In total, 27.8% (45/162) of the mothers were positive for anti-*N. caninum* IgG, among which antibodies were detected in the colostrum in 46.7% (21/45). All the ewes with positive colostrum had reactive offspring. The kappa agreement for the correlation between the serological tests on the ewes and the colostrum results was 0.558. This correlation increased as the antibody titers of the mothers increased, and reached 1.000 from the titer of 1:400 from the mothers. Comparison of the antibody detection results between the offspring’s serum and colostrum showed a kappa agreement of 1.000. In conclusion, there was a good agreement regarding the detection of anti-*N. caninum* IgG between the colostrum samples and the lambs’ serum; the use of colostrum forms a noninvasive alternative for diagnosing *N. caninum* in sheep herds.

## 1. Introduction

Neosporosis is a disease caused by the protozoon *Neospora caninum* (Apicomplexa: Sarcocystidae), which mainly causes reproductive problems such as miscarriage in intermediate hosts. However, it can also give rise to neurological symptoms in cases of congenital infection of neonates [1] and can form viable cysts in tissues. Under suitable conditions of low immunity, the latter may undergo recrudescence and can cause clinical signs in animals [1,2,3].

*N. caninum* needs to be controlled in order to prevent infection. Once an individual has become exposed to this protozoon, the infection may persist for the entire lifetime. Sheep can acquire the infection before birth, via the placenta [2,4]. This can cause significant damage to their fetuses, since the syndesmochorial nature of their placenta does not allow passage of antibodies. In this way, the transfer of maternal immunity to the offspring only occurs after ingestion of colostrum, which contains antibodies [5].

Studies have shown that *N. caninum* can have major impacts on pregnancy in sheep. Infection in the first and second trimesters of pregnancy causes high abortion rates, while in the final trimester, it can cause premature, infected, weak or unhealthy births [6].

Sheep farming stands out as an important livestock activity and has numerous advantages, such as needing a smaller breeding area, less forage consumption, ease of handling and production of multiple products from the same animal, such as meat, milk and good-quality leather [7].

Because sheep herds are mainly dedicated to the production of meat and leather, reproductive diseases can cause great damage; thus, leading to losses for producers. However, the economic importance of *N. caninum* infections in sheep is not fully understood [8]. Neosporosis is of great importance in small ruminants, as it is a disease that leads to the formation of cysts that can undergo reactivation under conditions of low immunity. Such conditions commonly occur during pregnancy and lead to abortion [9,10,11,12].

Therefore, taking into account the impacts that *N. caninum* can cause in sheep herds, the aim of this study was detect the correlation of IgG antibodies against *N. caninum* between serum and colostrum samples from ewes, and to evaluate the presence of this immunoglobulin in the serum of newborn lambs after the ingestion of colostrum.

## 2. Materials and Methods

### 2.1. Experimental Design

**Figure 1:**
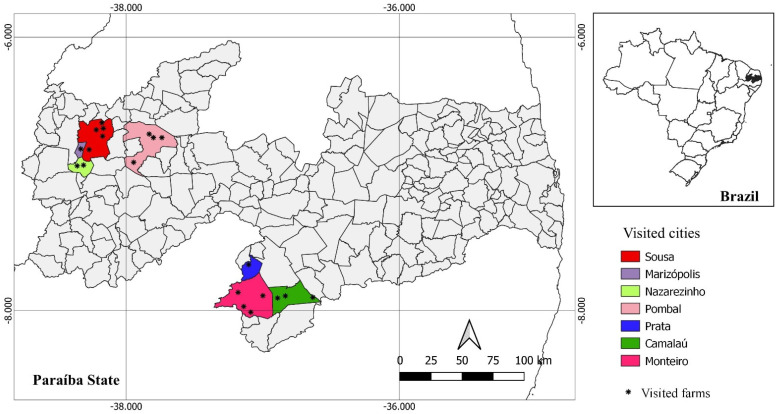
Geographical locations of the sheep herds analyzed in the semiarid region of the state of Paraíba, northeastern Brazil.

Twenty sheep breeding farms in the semiarid region of the state of Paraíba, Brazil, were selected according to convenience (Figure 1). All the visited farms had a history of abortions and premature births. During the study, 162 blood and colostrum samples were collected from ewes and 182 samples from their neonates (in cases of twin births, samples were collected from both lambs).

On each farm, ewes that were found to be healthy in a general physical examination were selected for serological and colostral samples to be taken. All of them had recently lambed and were not more than five days postpartum. Blood samples were also collected from the respective lambs, for the detection of anti-*N. caninum* and evaluation of passive immunization against this agent.

### 2.2. Blood and Colostrum Sampling

From each selected ewe, blood was collected by means of venipuncture of the jugular vein. Soon after this, colostrum was collected by manual milking. Simultaneously, blood samples were also taken from the newborn lambs of these selected ewes, between the second and fifth day after birth, to investigate the passive transfer of anti-*N. caninum* IgG. 

Colostrum was collected aseptically in sterile test tubes. The first three milk jets were always discarded in order to avoid contamination and then, approximately 5 mL of colostrum was collected from each ewe. Animals that presented clinical mastitis were not included in this study.

The blood samples were centrifuged at 1000× *g* for 10 min to obtain serum for analysis. The colostrum was also centrifuged and, after this procedure, the superficial fat layer (supernatant) was discarded and only the colostrum precipitate was used for the analyses. The blood serum and colostrum precipitate from each animal were properly identified and then, stored at −20 °C until the time of examination.

### 2.3. Serological and Colostral Tests

**Figure 2:**
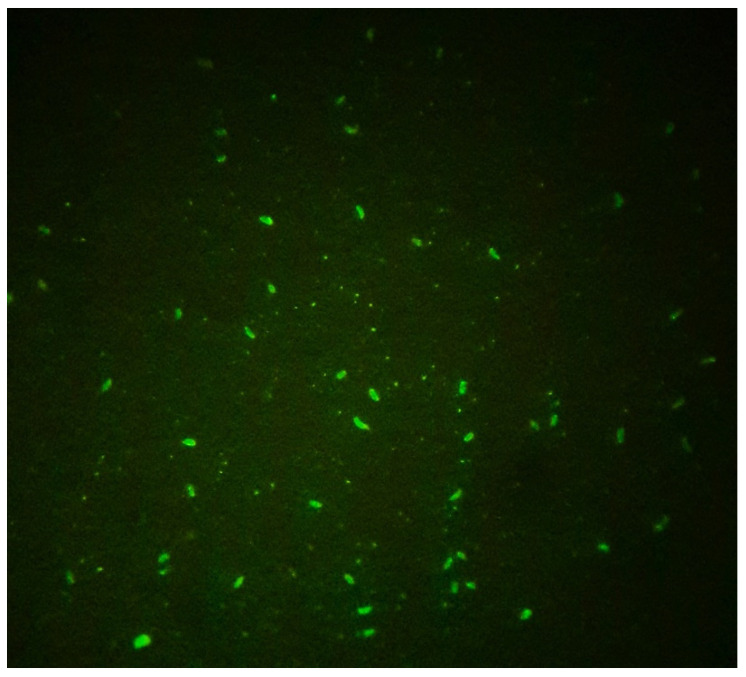
Positive sheep sample in the immunofluorescence antibody test (IFAT) for anti-*Neospora caninum* IgG, demonstrating total peripheral fluorescence of *N. caninum* tachyzoites visualized under ×40 objective (400× magnification).

The serum samples (ewes and lambs) and samples of colostrum precipitate were subjected to the immunofluorescence antibody test (IFAT) for anti-*N. caninum* IgG, always using the same protocol. As a complementary diagnostic, to avoid false positives, blood serum from all the sheep were submitted to IFAT for anti-*Toxoplasma gondii* IgG (Figure 2).

To the IFAT for anti-*N. caninum* IgG, the dilution used as a cutoff point for blood serum [13] and colostrum, adapted from Camillo et al. [14], was 1:50. The Nc-1 strain of tachyzoites, fixed on a slide, was used as the antigen [15]. The IFATs for anti-*T. gondii* IgG were performed according to Camargo [16]. The test was carried out using an RH strain of *T. gondii* tachyzoites fixed on a slide, with a cutoff point of 1:64 [17]. The analyses were carried out at the Laboratory of Immunology and Infectious Diseases (LIID) of the Instituto Federal da Paraíba, Sousa campus.

A whole-molecule sheep anti-IgG conjugate was used (SIGMA, St. Louis, MO, USA).

Serum and colostrum precipitate samples that reacted at dilutions greater than or equal to 1:50 were considered positive. The reactive serum and colostrum precipitates were titrated in sequential dilutions at base two until negative.

### 2.4. Statistical Analysis

A concordance analysis was performed on the results from the IFATs between serum samples from the ewes and their offspring and the colostrum samples using the kappa coefficient [18]. The sensitivity and specificity of the detection of antibodies in colostrum through IFAT were calculated in comparison with the detection of antibodies in the blood serum samples. To assess the sensitivity and specificity of the detection of antibodies in colostrum through IFAT, the detection of antibodies in the blood serum samples from the ewes and lambs was used as a standard [14].

## 3. Results

Among the 162 serological samples from the ewes, 27.8% (45/162) were positive for anti-*N. caninum* IgG, with titers of between 1:50 and 1:6400 (Table 1). All the ewes positive for anti-*N. caninum* antibodies were negative for anti-*T. gondii* antibodies.

It was observed that for 53.3% (24/45) of the ewes that were positive for anti-*N. caninum* in serum, no antibodies to this protozoon were detected in their colostrum. The titers for these ewes were between 1:50 and 1:200 (Table 1). Among all the ewes that had positive colostrum, their offspring were also reactive to anti-*N. caninum* antibodies. The ewes with titers greater than 1:100 showed higher possibilities of immunizing their offspring, except in cases of transplacental transmission where the offspring are born with anti-*N. caninum*.

There was a pattern among the samples that were positive in the IFAT. The serum samples from the ewes had levels greater than or equal to the levels of the colostrum titers, and the titers of their offspring were lower than or equal to the colostrum titers.

Moderate agreement was observed in the results from the IFATs on the ewes, in correlation with the results from colostrum, with a kappa coefficient of 0.558 (Table 2). It was also found that the titer results from the animals had an influence on the kappa coefficient results: the ewes with low titers showed results that were discordant with those from the respective colostrum, whereas the ewes with higher titers showed almost perfect kappa agreement (Table 2).

Comparison of the detection results regarding the anti-*N. caninum* levels between the lamb serum and colostrum showed a kappa agreement of 1.000.

## 4. Discussion

In this study, anti-*N. caninum* antibodies were found through IFAT in colostrum samples from the ewes evaluated. This is the first study worldwide that evaluated the detection of antibodies against *N. caninum* in colostrum from sheep and the agreement of IFAT regarding the serum antibody levels in ewes and lambs. A similar study was conducted by Ooi et al. [19] with regard to the detection of anti-*N. caninum* in the main fluids (serum, milk, vaginal secretion and saliva) from cattle through IFAT. High sensitivity was observed in that study; however, antibodies against the parasitic agent were more frequently detected in blood serum, followed by the milk of these animals. Meirelles et al. [20] investigated the detection of *T. gondii* in cows’ milk and observed that IFAT was not indicated for the detection of anti-*T. gondii* antibodies in this milk, since there was no agreement between blood serum and milk. Possibly the sensitivity observed in the present study was due to the fact that colostrum was used instead of milk.

Failure to pass on anti-*N. caninum* antibodies through colostrum was observed in the cases of 53.3% (24/45) of the ewes of the present study. Thus, for their offspring, passive immunization failed. One observed characteristic was that among these 24 ewes that did not have antibodies against *N. caninum* in their colostrum, 21 had a titer of 1:50. The kappa index observed in the analysis between serum from the ewes and their colostrum showed moderate agreement (0.558; Table 2). This may have occurred because the vast majority of the mothers evaluated that were positive in blood serum and negative in colostrum (21/24) had a serum titer of 1:50 (Table 1 and Table 2). This low titer may have meant that these animals did not have enough antibodies for their transfer to be detected in colostrum and in their offspring [21], such that they would have a positive result in the IFATs, which would have considerably reduced the level of agreement. Feitosa et al. [4] carried out a study evaluating the vertical transmission of IgG antibodies against *N. caninum* in naturally infected Santa Inês ewes. They observed that the ones with serum titers of 1:50 and 1:100 did not demonstrate good immunization of their offspring in the first month of life. In this regard, low titers of serum IgG in the mothers can influence the presence and concentration of anti-*N. caninum* in colostrum, which interferes with the passive immunization of their offspring.

Tamponi et al. [22] carried out a study in dairy sheep to evaluate the correlation between *N. caninum* antibodies through the ELISA method (ISCOM ELISA^®^ commercial kits, Uppsala, Sweden) in serum and milk samples from the same animals, where an excellent correlation was observed (r = 0.946; *p* < 0.001), with 96.45% of milk specificity compared to blood. The results found in our study may be related to factors such as the predominance of crossbred animals, production type, virulence of the strain circulating in the region and also, according to Wapenaar et al. [23], with the serum levels of antibodies, since naturally infected animals tend to have a significantly lower immune response [23].

In an experimental study on cattle, Cardoso et al. [24] used colostrum from cows that were positives for anti-*N. caninum* and observed that three of the eight suckled calves were not passively immunized against the parasite. This study differs from our study, in which a good passive immunization occurred in all the lambs that ingested the colostrum positive in IFAT, with IgG anti-*N. caninum* showing good passive immunization. In this case, there were several factors that might have led to the failure of the passive immunization of these calves through colostrum. It is known that the antibodies in colostrum are influenced by the levels of serum immunoglobulins, which are also produced in the udder. A reduction in antibody levels occurs rapidly over the first few weeks of lactation [25].

It was observed in the present study that the kappa agreement was higher among ewes with titers greater than or equal to 1:100. Similar results were observed among cattle by Camillo et al. [14] and Meirelles et al. [20], who found 100% agreement between blood serum and milk among cows with serum antibody titers above 1:100. This indicated that *N. caninum* in colostrum could be diagnosed through IFAT; however, with restrictions, especially among animals with titers of 1:50. In studies on outbreaks of abortions due to *N. caninum* infection, high titers were observed among those that suffered abortions. Thus, the detection of IgG in colostrum can be used as a screening test in these cases [26,27].

In the present study, the kappa index obtained in a comparative analysis between the lambs’ serum and colostrum showed a perfect agreement, regardless of the titer obtained from colostrum (1.000; Table 2). This was because the immunization of lambs only becomes possible if the colostrum presents enough anti-*Neospora caninum* IgG antibodies.

The analysis for diagnosing *N. caninum* demands specialized labor and a lot of time. Therefore, it can be suggested that, in sheep herds, IFAT should be performed on colostrum for diagnosing *N. caninum* in ewes. This makes it possible to identify the presence of this parasite in the herd and also makes it possible to ascertain whether passive immunization of the lambs has occurred, according to the titration. Therefore, when the colostrum presents titers greater than or equal to 1:100 and there was no failure of ingestion of colostrum by the lamb, this indicates that the probability of passive immunization of the offspring is extremely high, as demonstrated by the kappa index. It is well known that low titers are commonly associated with the initial phase of infections or with old infections, while higher titers indicate recent exposure to the agent [5,21].

Among cattle, excellent results have been demonstrated in studies evaluating the detection of anti-*N. caninum* in cow’s milk through IFAT. The main advantage of using this biological sample is that the material is collected noninvasively, which reduces the risks of disease transmission and high stress among the animals tested [14,20].

Among the ewes whose serum titers through IFAT were greater than or equal to 1:100, the concordance with the results from colostrum and from the lambs’ serum was greater. This allows the combined analysis of colostrum and the lambs’ serum, using only the serum or colostrum from adult females, given that seropositive females with titers of 1:100 will have similar titers in the colostrum. Consequently, these ewes’ offspring will be passively immunized. In this manner, analysis exclusively on serum or colostrum from ewes with titers greater than or equal to 1:100 can make the analysis more practical for herds.

## 5. Conclusions

It was concluded from the present study that there was a high level of agreement in the detection of anti-*N. caninum* antibodies between the serum of the ewes and their colostrum, especially with higher titers among the ewes. Moreover, there was a perfect agreement between the colostrum and the lambs’ serum. This makes it possible to use IFAT on colostrum as a noninvasive alternative for the evaluation of passive immunization of offspring from colostrum positive matrices and diagnosing *N. caninum* in sheep herds or mothers that present reproductive problems.

## Figures and Tables

**Table 1:** Detection and titration of anti-*Neospora caninum* IgG antibodies (Ab) by means of the indirect immunofluorescence reaction (IFAT) in blood serum and colostrum samples from ewes and in their lambs’ serum.

ID of Ewes and Lambs	Postpartum Days	Serum Titration of Ewes	Titration of Colostrum	Serum Titration of Lambs
2	4	200	200	100
3	3	3200	1600	100
5	3	200	100	50
6-L1	4	200	100	100
6-L2				50
9	2	200	-	-
10	5	50	-	-
11	2	200	-	-
12	3	50	-	-
18	4	50	-	-
21	2	50	-	-
27-L1	4	200	100	50
27-L2				50
28	2	50	-	-
29	2	50	-	-
31	3	100	100	100
34	3	6400	6400	100
35	4	200	200	200
37	2	50	-	-
38	2	100	50	50
39	4	50	-	-
42	4	50	50	50
61	2	100	50	50
63	2	200	-	-
64	2	400	400	400
71	3	50	50	50
81	4	50	-	-
82	3	50	-	-
83	2	50	-	-
101	5	50	-	-
103	5	100	100	100
107	4	50	-	-
108	3	50	-	-
112	3	50	50	50
113	3	50	50	50
115	4	100	50	50
123	4	50	-	-
125	4	50	-	-
126	3	50	-	-
127	3	50	-	-
128	2	50	-	-
134	5	50	-	-
135-L1	5	100	100	100
135-L2				100
140	5	50	-	-
146	5	50	50	50
147	4	100	50	50
148	2	50	50	50

L1: lamb 1; L2: lamb 2.

**Table 2:** Ewes that were positive and negative for anti-*Neospora caninum* IgG antibodies by means of the indirect fluorescence antibody test (IFAT) in colostrum and serum samples. Result stratified according to antibody titers obtained from the ewes’ serum, with comparison of the analyses on the ewes’ serum and colostrum according to the kappa coefficient.

Titer of the Ewes	Positive Ewes/Analyzed Ewes (%)	Positive Colostrum/Positive Ewes (%)	Kappa
1:50	27/162 (16.7%)	6/27 (22.2%)	0.323
1:100	7/162 (4.3%)	7/7 (100%)	1.000
1:200	8/162 (4.9%)	5/8 (62.5%)	0.760
1:400	1/162 (0.6%)	1/1 (100%)	1.000
1:3200	1/162 (0.6%)	1/1 (100%)	1.000
1:6400	1/162 (0.6%)	1/1 (100%)	1.000
Total	45/162 (27.7%)	21	0.558 *

* Kappa values: 0.20 to 0.40 indicates reasonable agreement; 0.40 to 0.60 indicates moderate agreement; 0.60 to 0.80 indicates substantive agreement; and 0.80 to 1.00 indicates perfect diagnostic agreement between the tests evaluated.

## Data Availability

Not applicable.
